# Incentive or Habit Learning in Amphibians?

**DOI:** 10.1371/journal.pone.0025798

**Published:** 2011-11-08

**Authors:** Rubén N. Muzio, Virginia Pistone Creydt, Mariana Iurman, Mauro A. Rinaldi, Bruno Sirani, Mauricio R. Papini

**Affiliations:** 1 Facultad de Psicología and Instituto de Biología y Medicina Experimental (IBYME-CONICET), Universidad de Buenos Aires, Buenos Aires, Argentina; 2 Department of Psychology, Texas Christian University, Fort Worth, Texas, United States of America; University of Western Ontario, Canada

## Abstract

Toads (*Rhinella arenarum*) received training with a novel incentive procedure involving access to solutions of different NaCl concentrations. In Experiment 1, instrumental behavior and weight variation data confirmed that such solutions yield incentive values ranging from appetitive (deionized water, DW, leading to weight gain), to neutral (300 mM slightly hypertonic solution, leading to no net weight gain or loss), and aversive (800 mM highly hypertonic solution leading to weight loss). In Experiment 2, a downshift from DW to a 300 mM solution or an upshift from a 300 mM solution to DW led to a gradual adjustment in instrumental behavior. In Experiment 3, extinction was similar after acquisition with access to only DW or with a random mixture of DW and 300 mM. In Experiment 4, a downshift from DW to 225, 212, or 200 mM solutions led again to gradual adjustments. These findings add to a growing body of comparative evidence suggesting that amphibians adjust to incentive shifts on the basis of habit formation and reorganization.

## Introduction

Do amphibians, an evolutionarily conservative lineage, encode information about appetitive stimuli (heretofore called incentives) or simply learn to select responses based on antecedent stimuli? Three situations involving shifts in incentive quality or magnitude have been extensively explored from a comparative perspective to determine whether incentive learning is required to explain vertebrate learning in general. These situations use widely spaced training conditions that avoid stimulus carry-over effects across trials (see [1]–[5]): (1) The postshift performance of animals shifted from a large to a small incentive, or vice versa; (2) The extinction performance of animals trained with either large or small incentives; and (3) The extinction performance of animals trained with either continuous or partial reinforcement (i.e., 100% or 50% trials ending in reinforcement). The two species of interest in this article, rats (*Rattus norvegicus*) and toads (*Rhinella arenarum*,  = *Bufo arenarum*), have been studied in these three situations and have produced evidence suggesting that whereas incentive encoding is necessary to explain mammalian learning, habit formation may be all that is needed to explain amphibian learning. The hallmark of habit formation is that the strength of behavior is directly related to the frequency and magnitude of the incentive (e.g., [6], [7]). Incentive learning, however, involves encoding some aspects of the incentive event that can then be anticipated (e.g., [8]), thus inducing emotional reactions when the expectation is violated, as in reward downshift situations (e.g., [9], [10]).

First, experiments with rats show that shifts in incentive quality or quantity induced changes in consummatory and instrumental behavior, relative to the behavior of unshifted controls (see [3], [9]). In the first demonstration of this effect, Tinklepaugh [11] reinforced one group of rats to reach a goal box in a complex maze with a preferred bran mash cereal, whereas a second group received sunflower seeds. A downshift from bran mash to seeds led to an increase in the number of errors, thus suggesting that rats had acquired an expectancy of bran mash and were reacting to its violation (i.e., incentive learning). This so-called successive negative contrast (SNC) effect does not always occur in experiments with rats. For example, rats reinforced with sucrose solutions for running down a runway exhibited no evidence of SNC in their instrumental behavior (iSNC), but they rejected the downshifted sucrose solution in the goal box, thus showing evidence of contrast in their consummatory behavior (cSNC) [12]. Results such as these suggest that cSNC may be more sensitive or easily triggered than iSNC. To accommodate this distinction, Papini and Pellegrini [13] (2006) argued that whereas cSNC is based on recognition memory (i.e., triggered by a failure to recognize the downshifted solution), iSNC is based on cued-recall memory (i.e., anticipatory reactivation of the preshift incentive). Papini, Muzio and Segura [14] reported that toads trained under conditions analogous to the runway procedure used with rats failed to exhibit iSNC. Instead, toads adjusted their postshift instrumental behavior to the current magnitude of the incentive without exhibiting contrast. Such a reversed iSNC effect implies habit formation, that is, a behavior mainly under the control of the current incentive magnitude.

Second, in rats, training with small rewards leads to greater resistance to extinction than training with large rewards (see [9]). This so-called magnitude of reinforcement extinction effect (MREE) has been reported in runways with different numbers of solid food pellets as the incentive [15], [16]. The MREE suggests that behavioral disruption is directly proportional to the magnitude of the discrepancy between anticipated and actual incentives, thus suggesting incentive learning. Toads trained under similar conditions produce a reversed MREE [14], [17]. Muzio, Segura and Papini [17] observed that groups of toads given access to water in each daily trial for a fixed amount of time (20, 80, 320, and 1,280 s) showed acquisition rates directly related to reward magnitude. Furthermore, asymptotic running latencies in acquisition were a monotonic function of actual water uptake (toads rehydrate by absorption through a specialized area of vascularized ventral skin located between the rear legs, known as pelvic patch, hence referred to as water uptake [18]). Water uptake was shown to be similar for the 20-s and 80-s conditions, and significantly lower than for the 320-s condition, which in turn was significantly lower than for the 1,280-s condition. However, despite experiencing differential incentive magnitudes in acquisition, when shifted to extinction toads reduced their instrumental behavior faster after being reinforced with small incentives than after acquisition with larger incentives. This reversed MREE suggests habit formation because the strength of behavior during extinction is directly related to the magnitude of the incentive during acquisition.

Third, with respect to extinction after partial vs. continuous reinforcement training, rats routinely produce the partial reinforcement extinction effect (PREE), namely, greater resistance to extinction after training with partial reinforcement rather than continuous reinforcement (see [9]). The spaced-trial PREE occurs in rats exposed to a wide variety of training parameters. Because partial reinforcement involves extensive exposure to expectancy violations during acquisition training, the PREE suggests incentive learning. In toads, however, partial reinforcement leads to weaker extinction performance than continuous reinforcement [17], [19]. For example, Muzio, Segura and Papini [19] reported that toads trained to traverse a runway for an opportunity to sit on a pool of deionized water for a fixed time interval acquired the approach response faster and later extinguished more slowly than toads exposed to an unsignaled mixture of reinforced and nonreinforced trials during acquisition. This is referred to as reversed PREE. Importantly, in reinforced trials, toads in both conditions uptake similar amounts of fluid. Thus, this reversed PREE in toads cannot be explained in terms of differential consumption of the incentive. The reversed PREE implies that extinction performance is controlled by habit formation because it is a direct function of incentive frequency in acquisition. (For a summary of the acronyms used in the text, see Table 1.)

**Table 1:** Acronyms used in the text.

Acronym	Full name and description
cSNC	*Consummatory successive negative contrast*: Lower consummatory behavior to a small incentive after training with a large incentive, relative to a condition always trained with the small incentive.
iSNC	*Instrumental successive negative contrast*: Lower performance searching for a small incentive after training with a large incentive, relative to a group always trained with the small incentive.
MREE	*Magnitude of reinforcement extinction effect*: Faster extinction after training with a large incentive than with a small incentive.
PREE	*Partial reinforcement extinction effect*: Slower extinction after training under partial incentive conditions than under continuous incentive conditions.
SPC	*Successive positive contrast*: Higher performance to a large incentive after training with a small incentive, relative to a condition always trained with the large incentive.

The present experiments further explore the effects of incentive shifts on behavior while adding some changes to the original training conditions. The first change is that a different lower reward condition was adopted here. Instead to arriving at a goal box with inaccessible deionized water as in previous experiments (both in extinction and in nonreinforced trials during partial reinforcement training), toads in the present experiments had access to a slightly hypertonic saline solution adjusted to minimize both water absorption and water loss. Previous studies have shown that toads have a plasma concentration that is approximately 245 mOsm/kg, which is isotonic to a 115 mM NaCl solution [20], [21]. A 300 mM is slightly hypertonic saline solution does not easily yield absorption or loss of water, thus tending to be motivationally neutral [22]. The neutrality of such a solution was assessed by measuring changes in body weight during each daily trial. In addition, the dependent variables were broadened by incorporating measures that had the potential to detect consummatory changes, including time in contact with the water, rubbing behavior (lateral movements of the toad's pelvis during water contact), and variations in ventral skin red coloration. The latter variable is associated with high blood supply observed during hydration in the pelvic patch. Unpublished observations indicated that these variables reflect changes correlated with water uptake [22]. Finally, both downshifts and upshifts in incentive magnitude were implemented. Still, the main goal of these experiments was to determine whether consummatory measures may detect the effects of incentive shifts on instrumental behavior, as it happens in rats trained with sucrose solutions (e.g., [12]).

## Experiment 1

The purpose of Experiment 1 was to test the effects of three different incentives on runway performance. Independent groups of toads were reinforced with solutions that either allowed for rehydration (deionized water), caused dehydration (800 mM highly hypertonic NaCl solution), or yielded little evidence of either rehydration or dehydration (300 mM slightly hypertonic NaCl solution). It was expected that these solutions would have, respectively, appetitive, aversive, and neutral incentive value. Thus, only deionized water was predicted to lead to improved runway performance. Daneri, Papini, and Muzio [23] reported that immersion in 800 mM saline solution and the resulting dehydration were sufficiently aversive to support anticipatory changes in heart rate and avoidance behavior.

### Methods

#### Subjects

The subjects were 18 experimentally naive, adult, male toads, *Rhinella arenarum* ( = *Bufo arenarum*), captured in ponds around Buenos Aires, Argentina. This species is not listed as threatened [24]. Animals were maintained according to the guidelines outlined by the NIH Guide for the Care and Use of Laboratory Animals. Upon arrival at the laboratory, toads were placed in cages (30 cm long, 21 cm wide, and 21 cm high) with a maximum of 10 animals per cage, where they remained with running tap water during at least two weeks. During the first week, subjects were treated with antibiotics and antiparasites mixed with insectivorous bird ground food (aproximately 3 g per day per animal). Then, animals were fed only with insectivorous bird ground (once a day during the second week, and after that, once a week). The vivarium was kept at a temperature between 21 and 23°C, and under a 16:8 h light∶dark cycle (light from 03:00 to 19:00 h). Before the start of the experiment, animals were transferred to individual cages with ad libitum deionized water. At the start of training toads were experimentally naive. Standard weights (weight of the hydrated animal after the urinary bladder has been emptied; [25]) varied between 97.6 g and 141.7 g, and did not differ significantly across groups, *F*(1, 12) = 1.94.

#### Apparatus

A runway was built with black Plexiglas and was divided into a start compartment (20 cm long), an alley (60 cm long), and a goal compartment (20 cm long). It was 12 cm wide and 20 cm high, and it was tilted (5°) so that the animal moved upward from the start to the goal compartment. Guillotine doors controlled the entrance to the alley from the start compartment, and the entrance to the goal box from the alley. In each section, a light bulb (15 W) provided diffuse illumination. A green Plexiglas container (13 cm long, 10 cm wide, and 3 cm high) with deionized water or different saline solutions was placed in the goal compartment. The container was filled with fluid, but the accessibility to the fluid was controlled by adjusting the height of a metallic grid placed in the container, which covered its entire surface and served as substrate. When the grid was put under the surface of the fluid, animals could reach the reward, but when the grid was placed above the surface, toads could not reach the fluid (this method was used during extinction trials). The runway was covered with translucent Plexiglas lids that allowed constant observation of the animals through a mirror. Training was carried out in a room where temperature (21–23°C) and humidity (48–52% HR) were controlled, and with constant background white noise (20–30,000 Hz).

#### Procedure

Animals received three 10-min pretraining sessions. In the first, drops of water were scattered about the floor in the alley and goal compartment. In the second session, drops of water were placed in the middle of the alley and right in front of the goal box. In the third, drops of water were placed right in front of the goal box. In all sessions, deionized water was placed in the goal container. Acquisition started the next day and lasted for 18 daily trials. Animals were divided into three groups (*n* = 6) differing in the reinforcer used in each trial. In Group DW, the container in the goal compartment contained deionized water; which allowed for rehydration and was thus considered to be an appetitive solution. In Group 300, the container in the goal compartment contained a 300 mM NaCl solution; such solution was slightly hypertonic with the internal milieu of the animal thus allowing for neither rehydration nor dehydration, thus considered a neutral solution (i.e., motivationally neutral). In Group 800, the container in the goal compartment contained an 800 mM NaCl solution; such solution was highly hypertonic, resulted in dehydration, and was thus considered an aversive solution [22].

Two dependent variables were recorded (following the same procedure used in all our previous studies; e.g., see [17]). (1) Running latency (in seconds): Time since the animal was completely within the alley and out of the start compartment, until it entered the goal compartment with its four legs. This variable has been used consistently from our first study on learning in toads as it proved to provide reliable data [17]. (2) Weight variation (g/100 g): Animals were weighed (in grams) before and after each trial to assess the amount of water uptake that occurred during the trial. The difference between these two weights was divided by the standard weight computed before the first pretraining session and multiplied by 100 to provide a relative measure of water uptake corrected for individual differences in body weight, as done in previous studies (e.g., [17]).

Trials were run between 12:00 and 19:00 h. Every day after the session, animals were transferred to dry cages where they remained until the next day. Toads were between 79% and 81% level of the standard weight at the start of each training trial. Toads were placed in the start box and after 30 s, the guillotine door was raised and the trial started. The running latency measure was recorded by the manual operation of a digital timer (1 s units) and transformed to the log_10_ to improve normality and allow for parametric statistics. Each animal was allowed a maximum of 300 s to leave the start compartment. Furthermore, each animal was allowed a maximum of 180 s to enter the goal compartment if already in the alley. A maximum latency of 180 s was assigned when (1) the animal failed to leave the start compartment after 300 s, or (2) the animal left the start compartment before the maximum 300 s, but failed to enter the goal compartment within the following 180 s. In incomplete trials, the animal was gently guided to the goal compartment where it received the outcome scheduled for that particular trial (guided trial). Analysis of variance (ANOVA) with trials as a repeated-measure factor whenever applicable, followed by pairwise comparisons of groups based on the Least Significant Difference (LSD) test were applied to all the data reported in this article. The alpha value was set to the 0.05 level for all tests.

### Results

**Figure 1:**
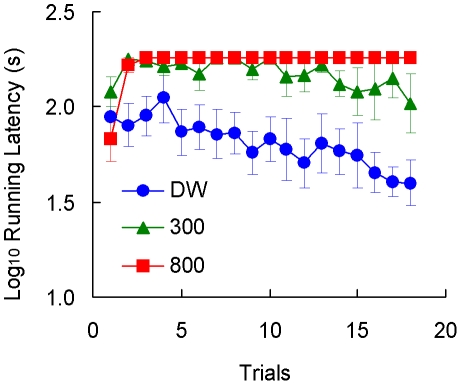
Runway performance in Experiment 1. Three groups of toads received deionized water (DW), 300 mM NaCl solution (300) or 800 mM NaCl solution (800) as reinforcers. Means ± standard errors (alpha = 0.05) are plotted.

**Figure 2:**
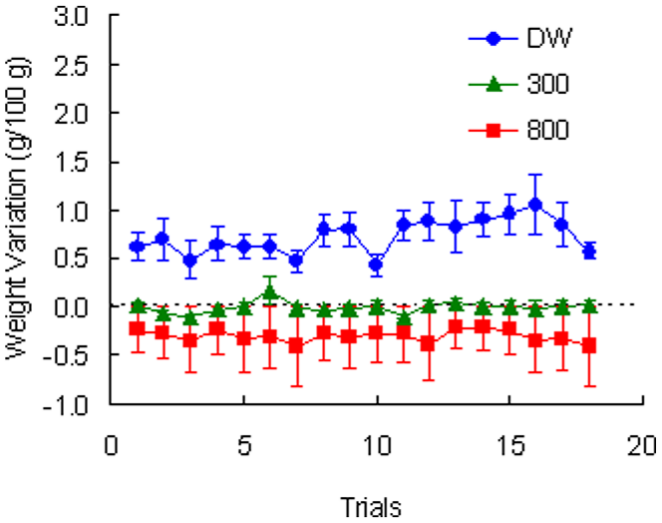
Weight variation in Experiment 1. Variations in body weight were corrected for individual differences in body weight across sessions. Means ± standard errors (alpha = 0.05) are plotted.

Toads in Group 800 rarely entered the goal box (see Figure 1). Because the procedure involved placing the animal at the goal when failing to complete the trial, the dehydration experienced at the goal effectively served as a punishing outcome suppressing instrumental approach. As shown in Figure 2, these toads actually lost weight as a result of these placements in an 800 mM NaCl solution.

The running latencies of the other two groups showed some change across trials, especially for Group DW (Figure 1). An analysis including only Groups DW and 300 provided the following results. For running latency, a Group (DW, 300) by Trial (1–18) analysis indicated significant group, *F*(1, 10) = 9.36, *p*<0.02, and trial effects, *F*(17, 170) = 3.57, *p*<0.001. The group by trial interaction was not significant, *F*(17, 170) = 1.25, *p*>0.23.

Figure 2 shows the relative change in weight across trials for the three groups of toads. The three incentives produced the expected changes in weight. Thus, DW allowed for weight gain during the trial, the 300 mM slightly hypertonic saline solution yielded little or no change in weight (i.e., motivationally neutral), and the 800 mM highly hypertonic saline solution led to dehydration. A Group (DW, 300, 800) by Trial (1–18) analysis produced a significant interaction, *F*(34, 255) = 1.80, *p*<0.01, as well as significant effects for group, *F*(2, 15) = 38.64, *p*<0.001, and trial, *F*(17, 255) = 2.26, *p*<0.01.

## Experiment 2

Papini et al. [14] reported that toads downshifted from a large to a small incentive magnitude exhibited a reversed iSNC. In those experiments, runway behavior was reinforced by access to deionized water for periods of either 1,280 s (large) or 80 s (small). Weight variation measured on a trial-by-trial basis demonstrated that the two rewards resulted in different amounts of body weight change, thus validating the magnitude manipulation. Toads also exhibited better performance when trained under the large reward than the small reward, thus showing that the two rewards had differential effects on behavior. However, the downshift manipulation only yielded evidence of an adjustment of behavior without contrast (i.e., a reversed iSNC effect). Similarly, whereas the unshifted large- and small-reward groups exhibited differential performance in acquisition, a shift to extinction yielded no evidence of an MREE.

The present experiment extended these treatments in the following ways. First, the small reward magnitude was a 300 mM slightly hypertonic solution (i.e., motivationally neutral). Experiment 1 showed that this slightly hypertonic solution results in no weight gain; thus, this solution provides similar sensory cues to those of water, without the motivational effect derived from water uptake. Unlike previous experiments [14], outcome duration was constant, but incentive magnitude was manipulated by adjusting the molarity of the solution. Second, this experiment included both downshifted and upshifted conditions, thus exploring the effects of negative as well as positive incentive shifts. There appears to be no information on successive positive contrast (SPC; see Table 1) in amphibians. Third, in addition to running latencies and changes in body weight, variations in the coloration of the pelvic patch were assessed.

### Methods

#### Subjects and apparatus

A total of 28 experimentally naive, adult, male toads served as subjects, obtained and maintained as described in Experiment 1. Toads received training in the same runway described in Experiment 1. An Agfa digital camera was used to photograph the ventral skin patch before and after each trial.

#### Procedure

Toads were randomly assigned to one of four groups (*n* = 7), each treated with a different reinforcement schedule. Training involved one trial per day, 7 days per week, at about the same time each day (from 10:00 to 16:00 h). Each toad received 12 preshift trials, followed by 12 postshift trials, and 12 extinction trials (a total of 36 daily trials). Animals in Group DW received deionized water on trials 1–24 (pre- and postshift trials). Animals in Group 300 received a 300 mM NaCl solution on trials 1–24. Animals in Group 300-DW received 300 mM NaCl solution on trials 1–12 (preshift) and deionized water on trials 13–24 (postshift). Animals in Group DW-300 received deionized water on trials 1–12 (preshift) and 300 mM NaCl solution on trials 13–24 (postshift). In all trials, whichever the outcome, toads spent 300 s in the goal box. In the following 12 days, toads received extinction trials. In these trials, toads could not reach the fluid in the goal box (as described in Experiment 1).

Three dependent variables were recorded. (1) Running latency (in seconds), as defined in Experiment 1. (2) Weight variation (g/100 g), also defined in Experiment 1. (3) Skin red coloration, defined as the redness intensity value for the central region of the pelvic patch. For each pixel within this area of the pelvic patch, red (*r*), green (*g*) and blue (*b*) values in RGB color-space were extracted and measured with a script developed in Matlab 2007, and a single redness intensity value (*R*) was computed as follows:
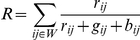
where *W* represents the region of interest in the pelvic patch. The *R* value, therefore, represents the normalized red component of the selected pixels, with larger values indicating redder patches. This value was used in previous studies to estimate the intensity of red coloration in other species [26]. After that, we calculated the *Skin color variation*, defined as the ratio between the coloration of the ventral skin patch after vs. before each trial. This red coloration index (*RI*) was computed as:
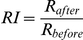
with *R_before_* and *R_after_*, the red coloration before and after each trial, respectively.

### Results

**Figure 3:**
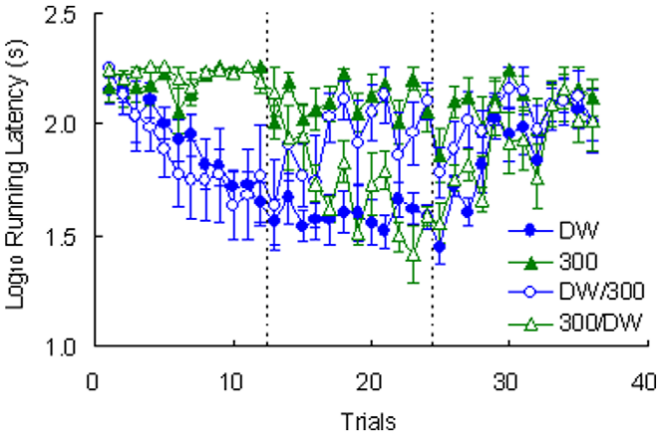
Runway performance in Experiment 2. Each group received in the preshift (12 trials) and then in the postshift (12 trials) the following reinforcers: deionized water and 300 mM NaCl solution (DW/300), 300 mM NaCl solution and deionized water (300/DW),only deionized water (DW), or only a 300 mM NaCl solution (300). Finally, all groups received an extinction phase (12 trials). Means ± standard errors (alpha = 0.05) are plotted.

Figure 3 shows the running latencies for the four groups. Preshift performance showed a clear differentiation of the two incentives. An Incentive (DW, 300) by Trial (1–12) analysis yielded significant effects for the interaction, *F*(11, 253) = 9.82, *p*<0.001, preshift incentive, *F*(1, 23) = 18.38, *p*<0.001, and trial, *F*(11, 253) = 6.88, *p*<0.001. Despite the differential control of performance by the incentives during preshift trials, there was no indication of contrast effects in these latencies, either positive or negative. A Preshift Incentive (DW, 300) by Postshift Incentive (DW, 300) by Trial (13–24) analysis of postshift data indicated significant double interactions between preshift incentive and trials, *F*(11, 231) = 3.54, *p*<0.001, and postshift incentive and trials, *F*(11, 231) = 4.49, *p*<0.001. However, the triple interaction failed to reach significance, *F*(11, 231) = 1.75, *p*>0.06, suggesting that the adjustment of shifted groups relative to their respective unshifted controls was accomplished at equivalent rates. There were also significant postshift differences, *F*(1, 21) = 22.83, *p*<0.001, and changes across trials, *F*(11, 231) = 2.22, *p*<0.02. Other effects were nonsignificant, *F*s<4.24, *p*s>0.051. Consistent with the absence of SNC, there was no indication of an MREE in the extinction data (i.e., no crossing over of extinction functions). A Group (DW, 300) by Trial (25–36) analysis indicated significant effects for the interaction, *F*(11, 132) = 2.31, *p*<0.02, across groups, *F*(1, 12) = 5.32, *p*<0.05, and across trials, *F*(11, 132) = 6.63, *p*<0.001.

**Figure 4:**
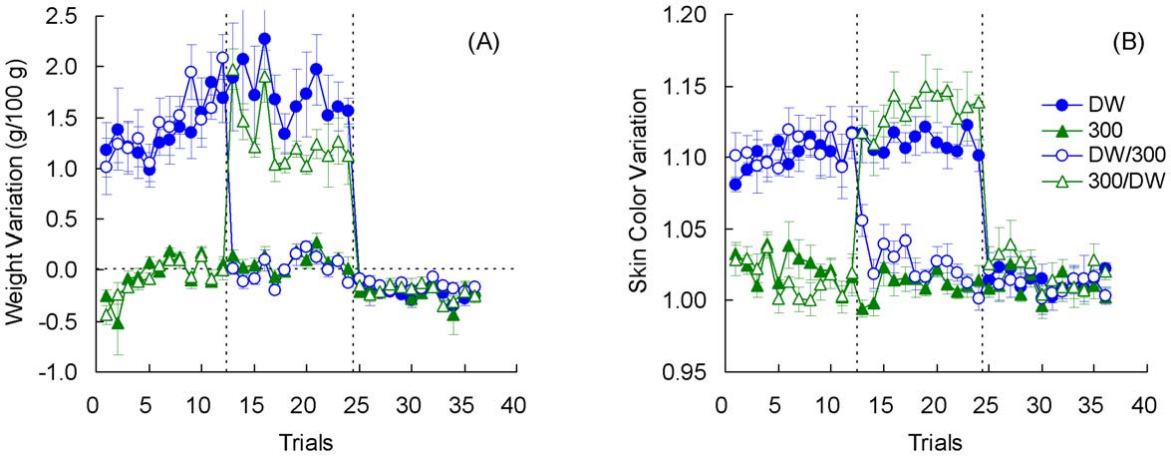
Weight variation (panel A) and Skin color variation (panel B) in Experiment 2. Means ± standard errors (alpha = 0.05) are plotted.

**Figure 5:**
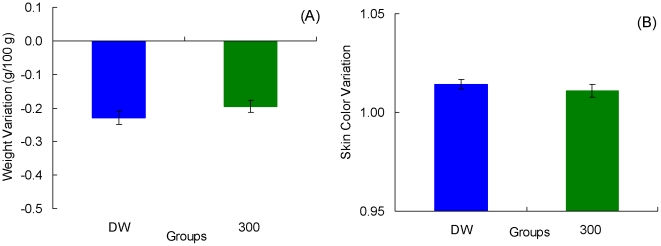
Extinction performance in Experiment 2. The results were averaged across trials for weight variation (panel A) and skin color variation (panel B) in groups DW and 300. Means ± standard errors (alpha = 0.05) are plotted.

Figure 4A shows the change in weights for each group. As observed in previous studies (e.g., [17]), toads improve their water uptake across trials. Preshift data were examined with an Preshift Incentive by Trial (1–12) analysis, which produced significant effects for all three factors, *F*s>3.54, *p*s<0.001. There was a tendency for upshifted toads to gain less weight than toads given always access to deionized water, but weights for the downshifted toads did not differ from their unshifted controls given only access to 300 mM saline solution. However, this trend was not confirmed by the statistical results. A Preshift Incentive by Postshift Incentive by Trial (13–24) analysis of postshift data indicated a significant postshift incentive by trial interaction, *F*(11, 231) = 2.67, *p*<0.004, as well as significant effects for postshift incentive and trial, *F*s>3.78, *p*s<0.001. Other factors were nonsignificant, *F*s<2.37, *p*s>0.13. A Preshift Incentive by Trial (25–36) analysis for extinction indicated only a significant reduction across trials (Figure 4A), *F*(11, 132) = 1.91, *p*<0.05; other factors were nonsignificant, *F*s<1.50, *p*s>0.24. Figure 5A shows the extinction results averaged across trials. Nonsignificant effects between groups were observed, *F*<1.

Figure 4B shows the variations in skin color on each trial. Preshift performance was significantly different across the two incentives, *F*(1, 23) = 399.89, *p*<0.001; the trial and incentive by trial interaction were nonsignificant, *F*s<1.39, *p*s>0.18. The postshift data show a trend for upshifted toads to exhibit greater variation in skin color than their unshifted controls always exposed to distilled water; variation was similar in downshifted toads and their unshifted controls always exposed to the 300 mM saline solution. The analysis detected this trend in terms of a significant preshift by postshift incentive interaction, *F*(1, 21) = 15.71, *p*<0.002. There was also a significant difference among postshift incentive conditions, *F*(1, 21) = 480.39, *p*<0.001. All other effects were nonsignificant, *F*s<1.51, *p*s<0.13.

Further analyses were calculated to understand the source of the significant preshift by postshift incentive interaction described above. Separate analyses for each shifted group and its respective unshifted control provided the following results. Upshifted Group 300/DW exhibited significantly higher skin color variation than unshifted Group DW, *F*(1, 10) = 6.83, *p*<0.03; the trial and incentive by trial interaction were nonsignificant, *F*s<1. Downshifted Group DW/300 was significantly higher than its unshifted control, Group 300, *F*(1, 11) = 12.09, *p*<0.006. In addition, the difference between these groups decreased significantly across trials, as shown by a significant interaction, *F*(11, 121) = 1.93, *p*<0.05. No changes across trials were detected, *F*(11, 121) = 1.51, *p*>0.13.

Figures 4B and 5B show the extinction results for skin color variation. A Preshift Incentive by Trial (25–36) analysis yielded nonsignificant effects for all factors, *F*s<1.

## Experiment 3

In addition to the PREE, rats exhibit greater resistance to extinction when a continuously reinforced food schedule is combined with a partial punishment schedule. For example, rats traversing a runway and obtaining food in every trial, but electric shock (i.e., peripheral pain) in a random 50% of the trials display greater resistance to extinction than rats trained only with food [27]. This experiment was designed to provide evidence of resistance to extinction using two types of negative outcomes: immersion in 300 mM slightly hypertonic saline solution (mentioned above) and immersion in 800 mM highly hypertonic saline solution. Daneri et al. [23] observed that such a hypertonic solution is an effective aversive reinforcer for toads, supporting both autonomic conditioning and avoidance learning. Three groups were included in the present experiment. Group DW was exposed to deionized water in all acquisition trials, whereas Groups DW/300 and DW/800 were exposed to 300 mM and 800 mM saline on a random 50% of acquisition trials. Acquisition was followed by extinction training for all animals. From a procedural viewpoint, Group DW/300 was considered analogous to a typical partial reinforcement schedule in which trials ending with exposure to slightly hypertonic saline solution were functionally analogous to nonreinforced trials. Group DW/800, however, was considered analogous to a partial punishment condition (e.g., [27]) in which intermittent exposure to a dehydrating event (exposure to highly hypertonic saline solution) was equivalent to punishment.

### Methods

#### Subjects and apparatus

The subjects were 24 experimentally naive, adult, male toads. They were obtained from the same source and maintained as described in Experiment 1. The same instruments described in Experiment 1 were also used in this experiment, with the following additions. All trials were videotaped with a Panasonic Newvicon video camera. Tapes were played back on the same device. Behavioral scoring (see below) was carried out using the program EthoLog 2.2 for Windows [28]. The observer was blind to the training conditions.

#### Procedure

Toads were randomly assigned to one of three groups (*n* = 8) differing in terms of the reinforcement schedule used during acquisition. All animals received one trial per day, 7 days per week, at about the same time each day (from 10:00 to 16:00 h). Group DW received deionized water on each of the 22 acquisition trials. Group DW-800 received deionized water on 50% of the trials and 800 mM NaCl solution on the remaining trials; these two types of trials were mixed pseudorandomly, so that there were no more than two consecutive trials of any type and deionized water was used on the first and last trials. Group DW-300 received deionized water on 50% of the trials and 300 mM NaCl solution on the rest, presented as described for the previous group. Acquisition was followed by 10 extinction trials in which the reinforcer was withheld for all animals. In all trials, whichever the outcome, toads spent 300 s in the goal box.

Five dependent variables were measured. (1) Running latency, as defined in Experiment 1. (2) Weight variation, also as defined in Experiment 1. (3) Skin coloration, as defined in Experiment 2. (4) Contact time (in seconds), defined as the cumulative time during which the animal had its four limbs and its pelvic patch in contact with the solution in the goal box. (5) Rubbing behavior, defined as the number of rhythmic lateral movements of the pelvis during contact time with the solution. This behavior is a component of what Stille [29] called the “water absorption response”. Unpublished studies suggest that this behavior is also observed while toads are engaged in weight gain in saline solutions of different concentrations [22].

### Results

**Figure 6:**
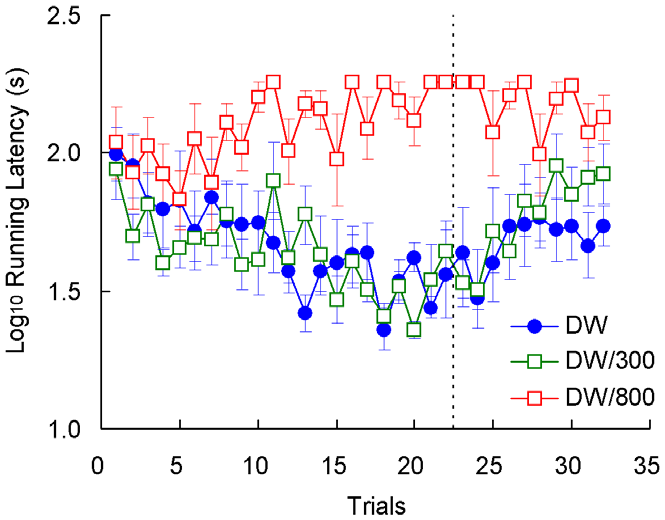
Runway performance in Experiment 3. Each group received in the preshift (22 trials) and then in the postshift (10 trials) the following reinforcers: deionized water and 300 mM NaCl solution (DW/300), deionized water and 800 mM NaCl solution (DW/800), or only deionized water (DW). Means ± standard errors (alpha = 0.05) are plotted.

**Figure 7:**
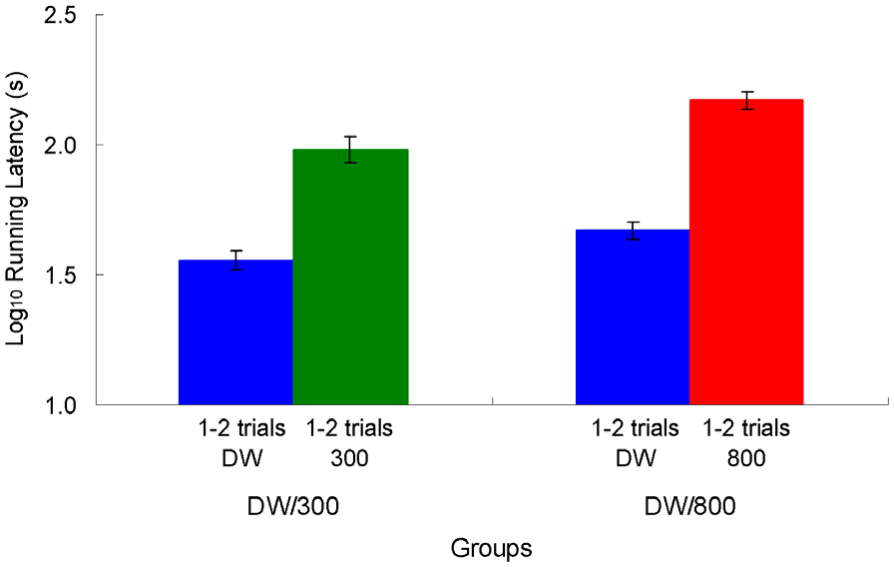
Reward following effect in Experiment 3. Average of running latencies after 1 or 2 trials reinforced with deionized water vs. 1 or 2 trials reinforced with a 300 mM NaCl solution (DW/300), or 1 or 2 trials reinforced with a 800 mM NaCl solution (DW/800). Means ± standard errors (alpha = 0.05) are plotted.

Running latencies are shown in Figure 6. For acquisition, the Group (DW, DW/300, DW/800) by Trial (1–22) analysis yielded significant effects for all three factors, *F*s>2.71, *p*s<0.001. LSD pairwise tests indicated that Group DW/800 was different from Groups DW and DW/300, *p*s<0.001, which in turn did not differ from each other, *p*>0.70. For extinction, the analysis indicated significant effects for all the factors, *F*s>1.79, *p*s<0.04, with the source being, again, Group DW/800, which differed from the other two groups, *p*s<0.003. Groups DW and DW/300 did not differ from each other, *p*>0.43. During acquisition, running latencies in Groups DW/300 and DW/800 changed according to the outcome of the preceding trial, exhibiting a reward following effect (Figure 7). Running latencies were significantly lower after 1 or 2 trials with deionized water than after 1 or 2 trials with (a) 300 mM NaCl solution, *F*(1, 126) = 4.83, *p*<0.05, or (b) 800 mM NaCl solution, *F*(1, 94) = 14.21, *p*<0.001.

**Figure 8:**
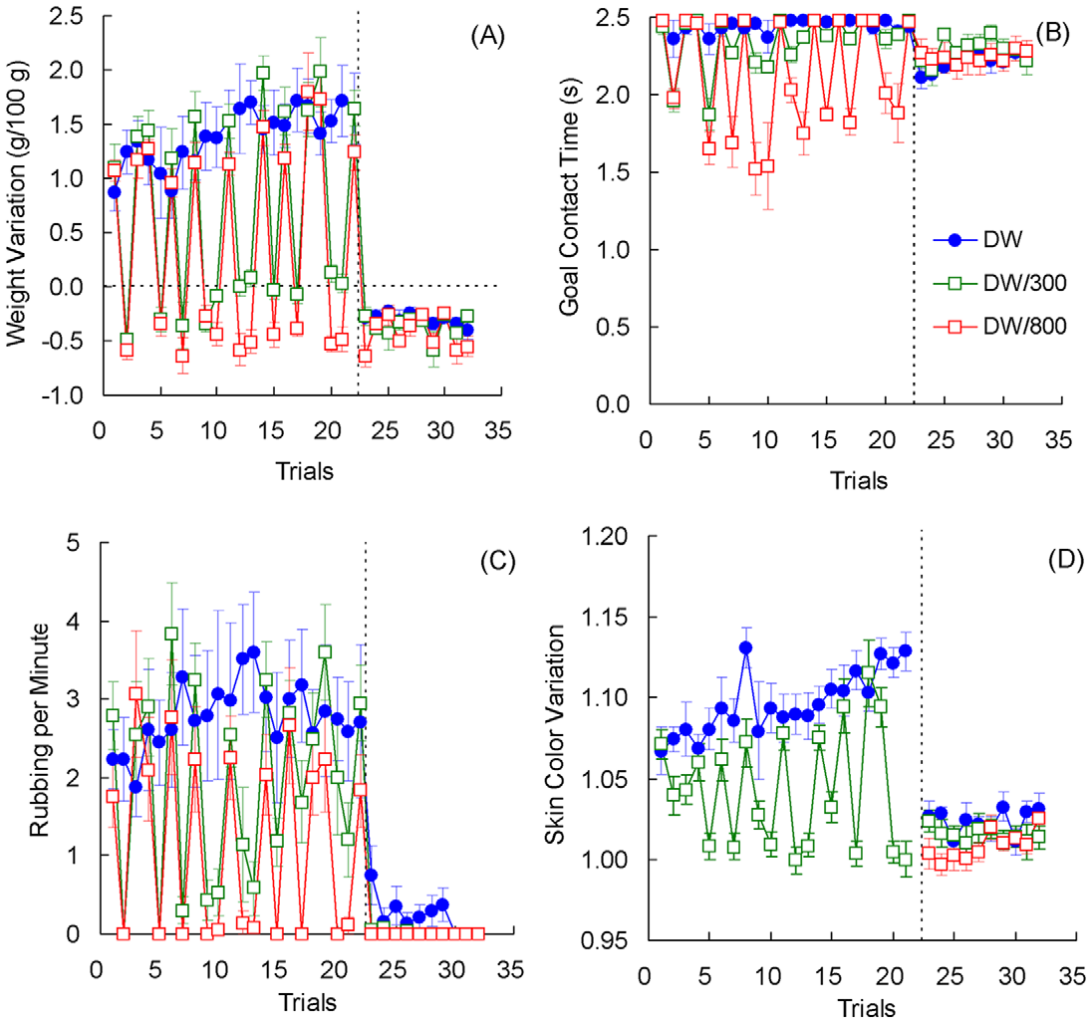
Results of Experiment 3. Weight variation (panel A), goal contact time (panel B), rubbing behavior (panel C), and skin color variation (panel D) in each of the three groups. Means ± standard errors (alpha = 0.05) are plotted.

**Figure 9:**
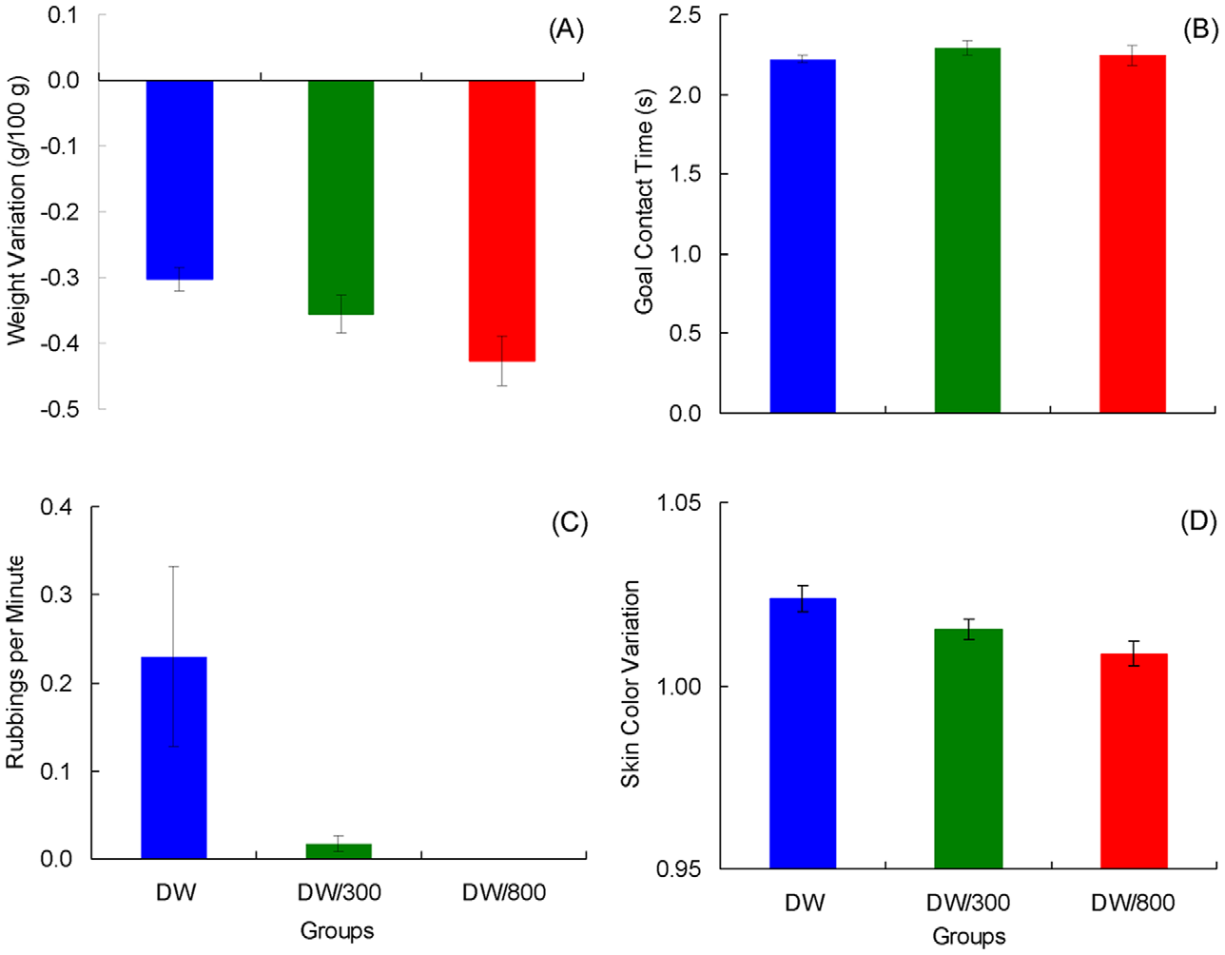
Extinction in Experiment 3. The results were averaged across trials for weight variation (panel A), goal contact time (panel B), rubbing behavior (panel C), and skin color variation (panel D) in each of the three groups. Means ± standard errors (alpha = 0.05) are plotted.

Weight variation data are presented in Figure 8A. This variable is directly related to water absorption or loss, thus providing an indication of the appetitive or aversive nature of the goal outcome. Given the drastic changes that occur as a function of whether or not toads have access to deionized water (reinforced trials), weights were processed according to two separate analyses for acquisition data. The first analysis involved all groups during reinforced trials (Trials 1, 3, 4, 6, 8, 11, 14, 16, 18, 19, and 22). As suggested in the figure, there was only a significant increase in weights across trials, *F*(10, 180) = 5.52, *p*<0.001, but nonsignificant group or interaction effects, *F*s>1.05, *p*s>0.37. The second analysis involved all groups during trials other than those included in the previous analysis (Trials 2, 5, 7, 9, 10, 12, 13, 15, 17, 20, and 21). This analysis showed significant differences across groups, *F*(2, 18) = 51.36, *p*<0.001, and trials, *F*(10, 180) = 2.71, *p*<0.005; their interaction was not significant, however, *F*(20, 180) = 1.10, *p*>0.35. As shown in Figure 8A, extinction data are compressed against the floor of the scale. Group differences can be better appreciated in Figure 9A, which shows the extinction performance averaged over trials 23–32. A Group by Trial analysis indicated a significant difference among groups, *F*(2, 18) = 4.47, *p*<0.03, and across trials, *F*(9, 162) = 2.40, *p*<0.02, but the interaction was not significant, *F*(18, 162) = 1.41, *p*>0.13. The group effect was attributable to the significant difference between Groups DW and DW/800, *p*<0.01; other pairwise effects were nonsignificant, *p*s>0.09.

The results for goal contact time are shown in Figure 8B. This variable provides a measure of consummatory behavior. An analysis for reinforced acquisition trials and for extinction trials indicated nonsignificant effects for all factors, *F*s<1.56, *p*s>0.13 (mean extinction data are also presented in Figure 9B). The analysis of the remaining acquisition trials indicated a significant interaction, *F*(20, 180) = 2.76, *p*<0.001, group effect, *F*(2, 18) = 38.42, *p*<0.001, and trial effect, *F*(10,180) = 5.66, *p*<0.001. Pairwise comparisons indicated that all groups differed from each other, *p*s<0.02.

Figure 8C shows the results in terms of rubbing responses, a behavior related to water uptake. An analysis of reinforced trials showed nonsignificant effects for all factors, *F*s<1.41, *p*s>0.18. For the rest of acquisition trials, the analysis indicated a significant interaction, *F*(20, 180) = 2.20, *p*<0.01, group effect, *F*(2, 18) = 11.37, *p*<0.002, and trial effect, *F*(10, 180) = 2.89, *p*<0.01. The source of the group effect was Group DW, which differed significantly from both Groups DW/300 and DW/800, *p*s<0.003, which in turn did not differ from each other, *p*>0.21. The extinction results (also shown in Figure 9C) indicated a significant interaction, *F*(18, 162) = 1.76, *p*<0.04, group effect, *F*(2, 18) = 4.62, *p*<0.03, and trial effect, *F*(9, 162) = 2.01, *p*<0.05. In extinction, Group DW exhibited a greater frequency of rubbing behavior than either Group DW/300 or DW/800, *p*s<0.02; the latter groups did not differ, *p*>0.84.

The last dependent variable to report was the change in skin coloration measured before and after each trial (Figure 8D). Toads in Group DW/800 exhibited strong skin irritation a few minutes after exposure to the highly hypertonic 800 mM saline solution and thus their acquisition data were excluded from the analyses. During extinction, there was no exposure to the aversive solution and, therefore, this effect was absent, thus allowing comparisons between the three groups. A camera malfunction resulted in the lost of all data corresponding to trial 22. A Group (DW, DW/300) by Reinforced Trial (1, 3, 4, 6, 8, 11, 14, 16, 18, and 19) analysis indicated only a significant change across trials, *F*(9, 117) = 4.79, *p*<0.001. Other *F* values were nonsignificant, *F*s<3.29, *p*s>0.09. A similar analysis of the remaining trial yielded significant effects for the interaction, *F*(10, 130) = 3.98, *p*<0.001, and the group effect, *F*(1, 13) = 69.53, *p*<0.001, but not for the trial effect, *F*(10, 130) = 1.49, *p*>0.14. Figures 7D and 8D show the results for color variation during extinction, for the three groups. An analysis indicated a significant difference among groups, *F*(2, 18) = 5.12, *p*<0.02, but nonsignificant effects for the other factors, *F*s<1. LSD comparisons indicated that the source of the group effect was a significant difference between Groups DW and DW/800, *p*<0.01; other pairwise comparisons were not significant, *p*s>0.07.

The results of Experiment 3 support several conclusions. First, there was no evidence of a PREE or even a reversed PREE, as Groups DW and DW/300 did not differ in extinction. Second, perhaps the most surprising result is that exposure to a highly hypertonic saline solution on 50% of the acquisition trials completely prevented the development of runway performance in Group DW/800. Moreover, in extinction trials, when toads did not have access to water, Group DW/800 actually dehydrated significantly more, exhibited significantly less rubbing behavior, and showed less variation in skin coloration than Group DW. These findings suggest that exposure to cues associated with dehydration have consequences similar to exposure to the highly hypertonic solution. Thus, these extinction effects are consistent with the conditioning findings reported by Daneri et al. [23] described above.

## Experiment 4

The incentive downshift manipulation introduced in Experiment 2 involved 300 mM saline solution, which produced little, if any, dehydration. Experiment 4 explored three different molar concentrations in an attempt to identify a mildly appetitive solution that might be functionally equivalent to a small incentive. Three concentrations were tested in separate experiments: 225, 212, and 200 mM NaCl solutions. In each case, toads were downshifted from deionized water and there was an unshifted control with the appropriate NaCl solution concentration.

### Methods

#### Subjects

A total of 38 experimentally naive, adult, male toads served as subjects. They were obtained from the same source, and maintained in the same manner described in Experiment 1.

#### Apparatus

The experimental device was a two-chamber black Plexiglas box (each chamber being 15×15×20 cm, L×W×H) as a start and a goal compartment. Goal compartment was connected to a hydraulic system that allowed for the presentation and draining of the appropriate solutions during the trial. The chambers were separated by a guillotine door and a barrier (15×3 cm, L×H). Toads were required to cross over the barrier with the four legs, moving from the start compartment to the goal compartment. The chambers were covered with translucent Plexiglas lids. The experimenter recorded the latency response by direct observation via a mirror positioned above the chambers.

#### Procedure

Three separate experiments were run each with naive toads and at different times; therefore, the results will be analyzed separately. These experiments differed in terms of the molarity of the downshifted incentive. In all three experiments, toads were downshifted from deionized water to 225 mM saline solution (Experiment 4a), to 212 mM saline solution (Experiment 4b), or to 200 mM saline solution (Experiment 4c). All toads received two 5-min trials (one per day) of pretraining. During these trials, the animals were free to move about in the experimental chambers. No stimuli were presented during these two pretraining trials. Afterward, animals were randomly assigned to the experimental or control group. Training started the following day. In the three experiments, toads received 12 daily preshift trials. In Experiments 4a and 4b, toads received 4 postshift trials, but in Experiment 4c the number of postshift trials was increased to 10 in an attempt to detect evidence of iSNC. Other aspects of the training procedure were as described in Experiment 2.

Two dependent variables were recorded. (1) Latency of response (in seconds), defined as the time elapsing from the start of the trial until the moment the animal was completely out of the start compartment and it entered the goal compartment with its four legs. (2) Weight variation, as defined in Experiment 1.

### Results

**Figure 10:**
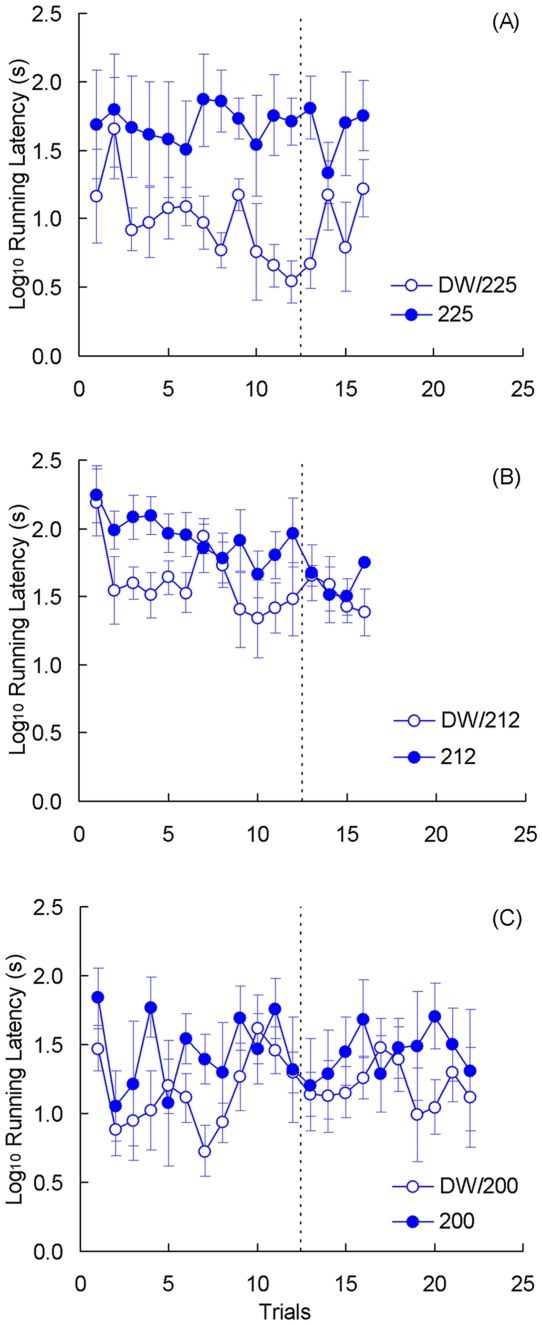
Instrumental performance in Experiment 4. Latencies responses during preshift (12 trials) and postshift (either 4 or 10 trials) of experimental groups of toads downshifted from deionized water to 225 mM NaCl solution (DW/225; panel A, Experiment 4a), to 212 mM saline solution (DW/212; panel B, Experiment 4b), or to 200 mM saline solution (DW/200; panel C, Experiment 4c), and its respective control groups (225, 212, or 200). Means ± standard errors (alpha = 0.05) are plotted.

**Figure 11:**
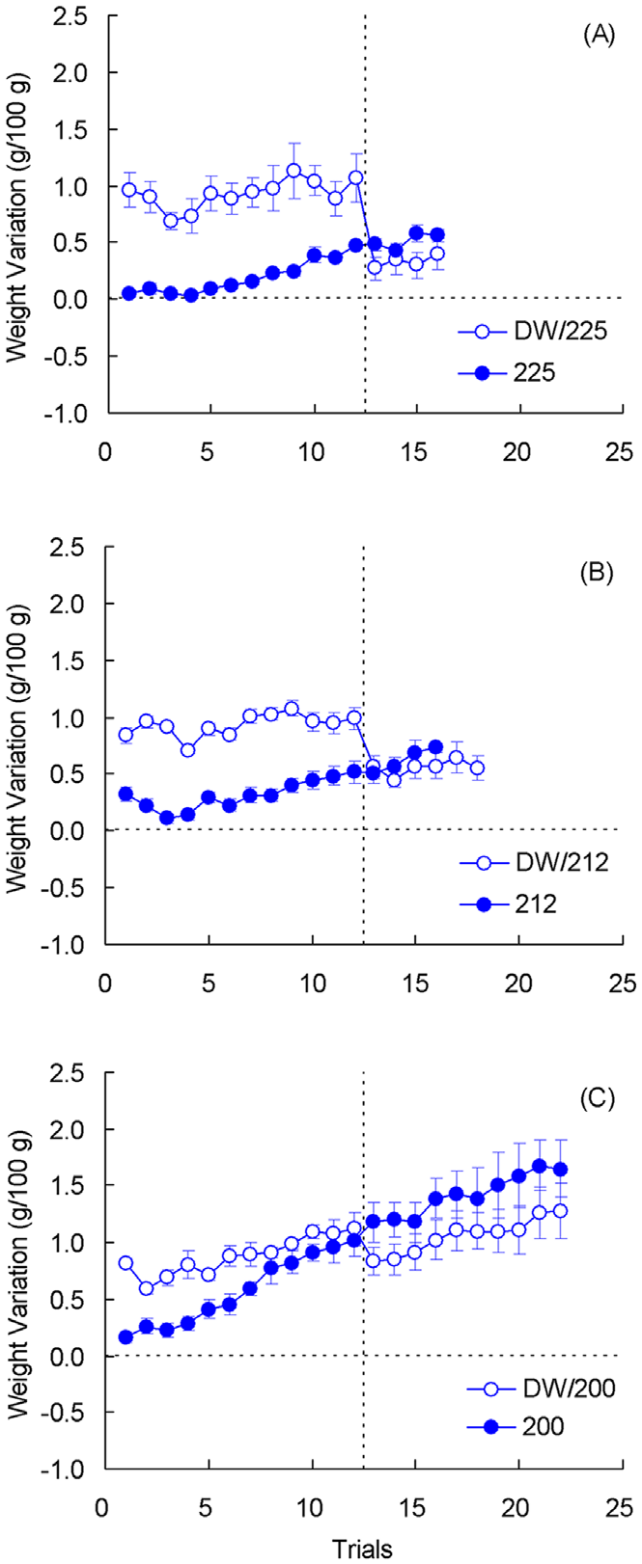
Weight variation in Experiments 4. Data from Experiment 4a (panel A), 4b (panel B), and 4c (panel C). Means ± standard errors (alpha = 0.05) are plotted.

For these three experiments, Group by Trial analyses were computed separately for each dependent variable (latency and weight variation) and for each phase (preshift and postshift). Figure 10A shows the behavioral results for Experiment 4a. The analysis of preshift performance showed a significant group difference, *F*(1, 10) = 12.31, *p*<0.01, but nonsignificant effects for trials or the group by trial interaction, *F*s<1. Although the three groups receiving access to distilled water (Groups DW/200, DW/212, and DW/225) were treated identically, their performance was not exactly the same. These differences are attributable to variations in the month of training and possibly in the type of pre-laboratory experience of individual animals. Postshift latencies provided no indication of cSNC. In fact, latencies for Group 225 were significantly higher than those for Group DW/225, *F*(1, 10) = 9.22, *p*<0.02. Other effects were nonsignificant, *F*s<1.55, *p*s>0.22. Figure 11A shows weight variations also for Experiment 4a. During preshift trials, there were significant effects for groups, *F*(1, 10) = 34.72, *p*<0.001, and trials, *F*(11, 110) = 4.89, *p*<0.001; their interaction was not significant, *F*(11, 110 = 1.09, *p*>0.37. The postshift results show a hint of contrast as the weight change was consistently lower for the downshifted toads than for the unshifted controls. However, only the change across trials was significant, *F*(3, 30) = 3.72, *p*<0.03. There were nonsignificant effects for groups, *F*(1, 10) = 2.13, *p*>0.17, and for the group by trial interaction, *F*(3, 30) = 2.75, *p* = 0.060.

Figure 10B shows the behavioral results for Experiment 4b. With a more diluted saline solution than in Experiment 4a (212 vs. 225 mM), toads exhibited improved performance during preshift trials. Thus, there was a significant reduction in latencies, *F*(11, 132) = 2.49, *p*<0.01. However, neither the group effect, *F*(1, 12) = 4.33, *p*>0.05, nor the group by trial interaction, F<1, reached significance. There was also no clear evidence of incentive contrast in the postshift performance, with none of the factors reaching significance, *F*s<1.10, *p*s>0.36. Figure 11B shows weight variations for these toads. During preshift trials, there significant differences between groups, *F*(1, 12) = 50.27, *p*<0.001, and across trials, *F*(11, 132) = 4.96, *p*<0.001; their interaction was not significant, *F*(11, 132) = 1.55, *p* = 0.120. Interestingly, weights change scores seemed to decrease more in Group DW/212 relative to Group DW. This was detected as a significant group by trial interaction, *F*(3, 36) = 3.98, *p*<0.02. Weights also changed significantly across trials, *F*(3, 36) = 8.67, *p*<0.001, but groups were not different from each other, *F*<1.

Figure 10C shows the behavioral effects for Experiment 4c. With a further reduction in the molarity of saline solution the preshift performance of the groups was very similar. The analysis showed only a significant change across trials, *F*(11, 110) = 1.97, *p*<0.04; no effects were found for the other factors, *F*s<2.46, *p*s>0.14. As in the previous experiments, no behavioral evidence of incentive contrast was found, even with an extended postshift phase (10 vs. 4 trials in the two previous experiments). None of the factors reached significant levels, *F*s<1. Figure 11C shows weight variations for these toads. In terms of weight variation, preshift trials indicated significant effects for all factors: group, *F*(1, 10) = 15.59, *p*<0.01, trials, *F*(11, 110) = 19.72, *p*<0.001, and their interaction, *F*(11, 110) = 2.77, *p*<0.01. Weight variations during postshift trials exhibited a clear tendency in the downshifted toads to score below the unshifted controls on average. However, only the trial effect was significant, *F*(9, 90) = 9.36, *p*<0.001. Other effects were not significant: groups, *F*(1, 10) = 1.75, *p*>0.21; interaction, *F*<1.

## Discussion

The results of the present experiments show that the salinity of the solution can be effectively used with terrestrial toads to introduce ecologically relevant appetitive and aversive stimuli. These incentives promote adaptive behavior in terms of approach to rehydrating solutions and avoidance and escape from dehydrating solutions. Interestingly, the evidence suggests toads achieve such adaptive responses via habit formation, rather than by encoding specific information about the incentive. Thus, there was no behavioral evidence of either positive or negative successive contrast (Experiments 2 and 4) or of a partial-reinforcement or partial-punishment extinction effect (Experiment 3). These effects are detected in terms of anticipatory behavior, that is, of instrumental behavior occurring before the organism is in actual contact with the incentive (Papini, 2003). As mentioned in the introduction, a variety of results suggest that the consummatory behavior of mammals may be more sensitive to incentive shifts than their instrumental behavior (e.g., [12]). Moreover, lesion experiments show that iSNC and cSNC are dissociable [30]–[32]. Accordingly, additional dependent variables were registered in an attempt to detect evidence for incentive encoding in toads, but none of them provided clear results. A hint of incentive encoding was obtained in Experiment 4 in terms of weight variation data. Albeit nonsignificant, there was a tendency for toads to rehydrate less when in contact with the downshifted solution than was the case in unshifted controls (see Figure 11).

There are two major interpretations of the present results. One interpretation is that the lack of evidence in favor of incentive encoding is a simple consequence of not having yet found the right parameters to reveal it. Accordingly, the experiment varies the conditions of training until some combination produces the desired effect. This procedure, called systematic variation [1], has one main problem: it does not specify how many variations are needed before one can be satisfied that the phenomenon in question is not present [33]. The data accumulated thus far suggest that the behavior of toads in situations involving shifts in incentive value can be described exclusively in terms of stimulus-response habits and without reference to incentive learning (e.g., [17]). A potential route of exploration involves treatments known to enhance these effects in mammals. For example, opioid receptor blockage with naloxone (a nonselective opioid receptor antagonist) and naltrindole (a delta-opioid receptor antagonist) has been shown to enhance cSNC in rats [34], [35]. Opioid receptors have been described in amphibians and are known to be homologous to those found in mammals [36], [37]. In addition, pretrial induction of inflammatory peripheral pain (e.g., with a formalin injection in a hind paw) also enhances cSNC in rats [38]. It remains to be determined whether analogous treatments in toads result in the emergence of incentive contrast effects.

A second interpretation suggests that the behavioral differences between toads and rats observed in situations involving incentive shifts reflect the evolution of learning mechanisms present in mammals, but either absent or not fully developed in amphibians. Papini [4], [5], [10] argued that all vertebrates share the ability to acquire information about changes occurring in their environment, a cognitive process termed allocentric learning. Allocentric mechanisms allow animals to adjust their behavior to the current conditions of incentive from whatever behavioral level was supported by the value of prior incentives. This mechanism yields the reversed effects described in the introduction -SNC, MREE, and PREE. Allocentric learning is also consistent with habit formation. When incentive changes are motivationally significant, vertebrates have the ability to react emotionally and to learn about their own reactions to the change. Such egocentric learning is general among vertebrates when it involves pain and fear, but exclusive to mammals when it involves incentive devaluations or omissions (for a review of the evidence, see [5]). Notice that egocentric learning requires incentive encoding, but goes beyond it in that a failure to recognize the anticipated outcome generates an emotional response of frustration that is, in turn, encoded and later anticipated when the organism is exposed to the appropriate situation [9]. The results reported in the present experiments can be explained exclusively on the basis of allocentric learning and are, therefore, consistent with the evolutionary divergence hypothesized on the basis of additional comparative research with vertebrates (see also [39]).

In conclusion, amphibians adjusted to shifts in incentives by gradual behavioral reorganization, rather than abruptly reacting to unexpected changes in incentive magnitudes, as it has been shown in mammals [10]. These findings add to a growing body of comparative evidence suggesting that relatively more conservative vertebrate lineages regulate their behavior predominantly on the basis of habit formation and reorganization.
